# Research trends and hotspots in prostate cancer associated exosome: a bibliometric analysis

**DOI:** 10.3389/fonc.2023.1270104

**Published:** 2023-11-21

**Authors:** Zhengjia Zhu, Yingjian Zhou, Hao Li, Wenchao Xu, Tao Wang, Jihong Liu, Hongyang Jiang

**Affiliations:** ^1^ Department of Urology, Tongji Hospital, Tongji Medical College, Huazhong University of Science and Technology, Wuhan, China; ^2^ Institute of Urology, Tongji Hospital, Tongji Medical College, Huazhong University of Science and Technology, Wuhan, China

**Keywords:** exosome, prostate cancer, bibliometric analysis, bone metastasis, tumor suppressor, drug resistance

## Abstract

**Background:**

Prostate cancer is viewed as the second most common cancer in men worldwide. In our study, we used bibliometric analysis to construct a visual map of the relationship between prostate cancer and exosomes with the intent of uncovering research trends and current hotspots in this field.

**Method:**

We searched the Web of Science Core Collection for all publications in the prostate cancer associated with exosome field came out since 2010. With the assistance of bibliometric analysis software such as VOSviewer and CiteSpace, we conducted data extraction and analysis for countries/regions, institutions, authors, journals, references and keywords.

**Results:**

A bibliometric analysis of 990 publications was performed. Since 2010, the published quantity and cited frequency of the prostate cancer-associated exosome field have revealed an increasing tendency. In this field, we visualized the research trends by the means of analyzing the references and keywords. We obtained the statistical data: the total citations of publications have increased to 55,462, the average citation per article has reached 55.3 times, and the H-index has amounted to 110. Our findings supported that USA, China and Italy rank the top countries with both the maximum publications and strongest cooperations. Harvard Medical School, Cedars Sinai Med Ctr, Johns Hopkins University, are top institutions in the center of research as they are held to be. Thery C, Skog J and Taylor DD are the leading and outstanding professors and researchers. And top journals like Prostate, Plos One and Journal of Extracellular Vesicle expressed keen interests in this field. Based on our analysis and research, we believe that this field is attracting more and more attention and will focus on tumor bone metastasis, drug delivery, and tumor suppressor.

**Conclusion:**

In the past 12 years, researchers have dedicated their efforts to prostate cancer associated exosome. On the basis of previous studies, scientists are showing increasingly solicitude for the role of exosome in prostate cancer progression and potential therapy such as drug delivery.

## 1 Introduction

Prostate cancer (PCa), with the highest incidence of human disease, impacts patients’ health condition and quality of life and remains the second leading cause of male mortality with an estimated 34,130 deaths in 2021 in the US (1). This makes the public pay attention to screening for prostate cancer to alleviate their anxiety about risk of cancer. Therefore, in the past decade, the content, principles, methods, safety, reliability, and efficiency of prostate cancer examination have also become a hot research topic in this field. Besides, adopting the most advanced examination methods so as to find potential PCa at early period and give accurate diagnosis is believed to be a useful way to improve cure rate.

Compared with rectal proctoscopy, Computed Tomography (CT) and Magnetic Resonance Imaging (MRI), biomarkers for PCa are less traumatic, more convenient in operation, and more economical and practical. During the tumor progression, a variety of bioactive substance will be released to the serum, which will lead to changes in concentration of these special substances. Regular serum prostate-specific antigen (PSA) evaluation and digital rectal examination (DRE) are recognized as most commonplace methods for detecting PCa (2). The serum PSA level is among the best of the screening tools available in medicine today and is viewed as the best marker for early detection (3). While PSA examination has little effect in distinguishing whether tumors are benign or malignant, so researchers aspire for the role of other small molecule biomarkers. In the tumor microenvironment, exosomes are considered to be diverse and carry various functions (4–8). The research on exosomes related to prostate cancer has gradually entered people’s vision.

Extracellular vesicles (EVs) are lipid-enclosed vesicles released by cells into the extracellular environment. The three main subtypes of EVs are microvesicles (MVs), exosomes, and apoptotic bodies, which are differentiated based upon their biogenesis, release pathways, size, content, and function. Exosomes are derived and secreted by different cells, containing specific RNA and protein which maintain bioactive functions (9–11). These small molecules can be released and uptaken by various target cells. The composition and cargo of exosomes vary from others owing to their derived cells. For this reason, exosomes are thought to be potential approach for examine PCa at early period. However, the identity and mechanism of exosomes still retain obscure. On the other hand, prior studies demonstrate that exosomes have traits to be effective therapeutic tool against prostate cancer (11–13). Since they are not only able to deliver medicine to specific target cells but also useful for enhancing the cytotoxic effect of drug. As a result, exosome mediated drug delivery is promising research which will be important for promoting the accuracy of examination and treatment of PCa.

In the past decade, research in this field has shown an upward trend, and more and more literature has emerged. Although there are a few systematic reviews summarizing recent research on exosome and the relationship between exosome and cancer, among others. However, there is no relevant quantitative analysis on the role of exosomes in prostate cancer. Bibliometric analysis is the process of analyzing a certain amount of literature to identify topics and popular citations that have been well researched in this field (14–16). It analyzes the focus that has gradually gained widespread attention in the development process of this field and helps researchers discover hot topics that can be explored and have not yet been studied (14–16). This study presents a visual, bibliometric and scientometric analysis based on the application of various literature analysis software, providing emerging hot research directions.

## 2 Method

### 2.1 Database

As widely recognized as one of the most reputable and comprehensive databases in the field of scientific research, WOSCC encompasses a vast collection of interdisciplinary literature and research (17). Consequently, for bibliometric studies, WOSCC is often regarded as the most suitable database for acquiring data (18, 19). For the purposes of this study, we conducted a thorough literature search utilizing the Web of Science Core Collection (WosCC).

### 2.2 Search strategies

Our search strategy was as follows: ((TS= (“exosom*”) OR TS= (“exosc*”) NOT TS= (“exoscreen”) NOT TS= (“exoscop*”) NOT TS= (“exosca*”)) AND (TS=(“prostat*” NEAR/1 “cancer*” OR “prostat*” NEAR/1 “tumor*” OR “prostat*” NEAR/1 “tumour*” OR “prostat*” NEAR/1 “oncology” OR “prostat*” NEAR/1 “neoplasm*” OR “prostat*” NEAR/1 “carcinoma*” OR “prostat*” NEAR/1 “adenocarcinoma*” OR “prostat*” NEAR/1 “adenocarcinoma*”))) (20, 21). The language of the literature was restricted to English. And the type of 92 literature was limited to articles and reviews. Literature from 2010 to 2022 was selected. All 93 information of the retrieved literature was saved in plain text format for analysis.

### 2.3 Data extraction & analysis

To eliminate duplication, the plain text files extracted from the WOSCC were imported into CiteSpace V (6.1.R2 Basic version, Drexel University, United States). Subsequently, the non-duplicate files were gathered and imported into Microsoft Excel for analysis of publication authors, countries, journals, and institutions. Additionally, data on publication countries, journals, authors, institutions, citation frequencies, and H-index were obtained from WOS on 17^th^ January 2023. VOSviewer was utilized to analyze and acquire the Total Link Strength (TLS), while CiteSpace was employed for centrality analysis.

### 2.4 Data visualization

In this study, we conducted bibliometric analysis and visualization using various tools including VOSviewer (version 1.6.18), CiteSpace V (version 6.1.R2 basic), Tableau, an online bibliometric analysis platform (bibliometric.com), and Microsoft Excel. VOSviewer is a widely utilized software for bibliometric analysis that facilitates visual representation of the relationships between different nodes and displays associated information through their characteristics (22). In our study, we employed VOSviewer to generate visualizations of co-authorship among countries, institutions, authors, journals, citation relationships of 109 references, and co-occurrence of keywords, resulting in concise and clear visual representations. CiteSpace, a Java-based bibliometric analysis software developed by Prof. Chaomei Chen (23), was chosen due to its outstanding performance in burst detection, centrality calculation, citation relationship visualization, and clustering analysis (24). Thus, we employed CiteSpace to visualize dual maps of journals, citation bursts of keywords or references, timelines, and authors’ co-citation relationships.

## 3 Results

**Figure 1:**
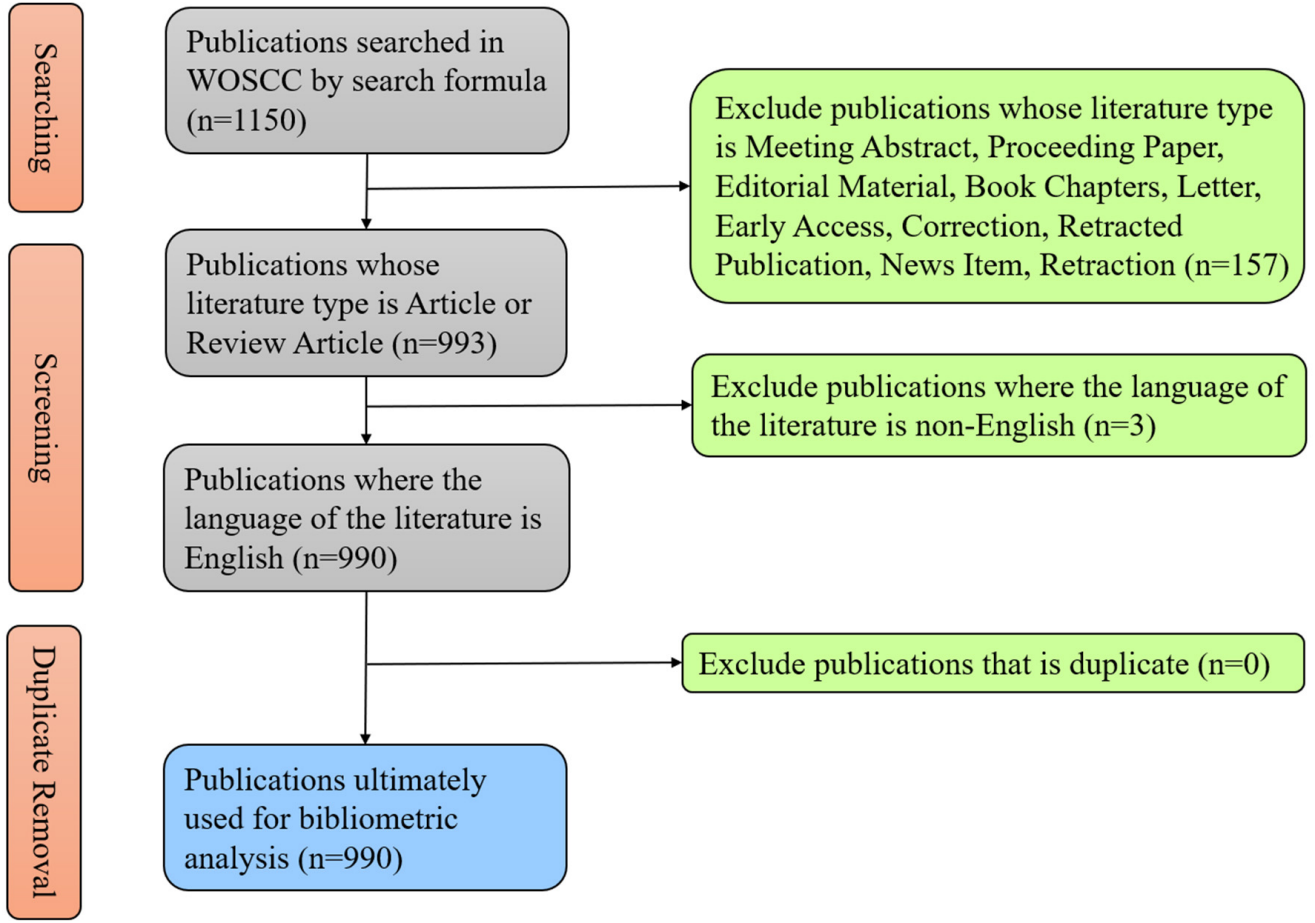
Shows the flow chart of publication acquisition, filtering and de-duplication.

According to the previous literature, we used a search formula to conduct a research about the literature and data in the field of exosome in prostate cancer on WoSCC and finally collected 1150 literatures published from 2010 to 2022. After restricting some conditions, we sifted out 990 publications to carry out following analysis (**Figure 1**). As of the search date, the total citations of publications have reached up to 55,462, with 55.3 times for average per item, and the H-index have reached 110.

### 3.1 Global trends in publication volume and citation frequency

**Figure 2:**
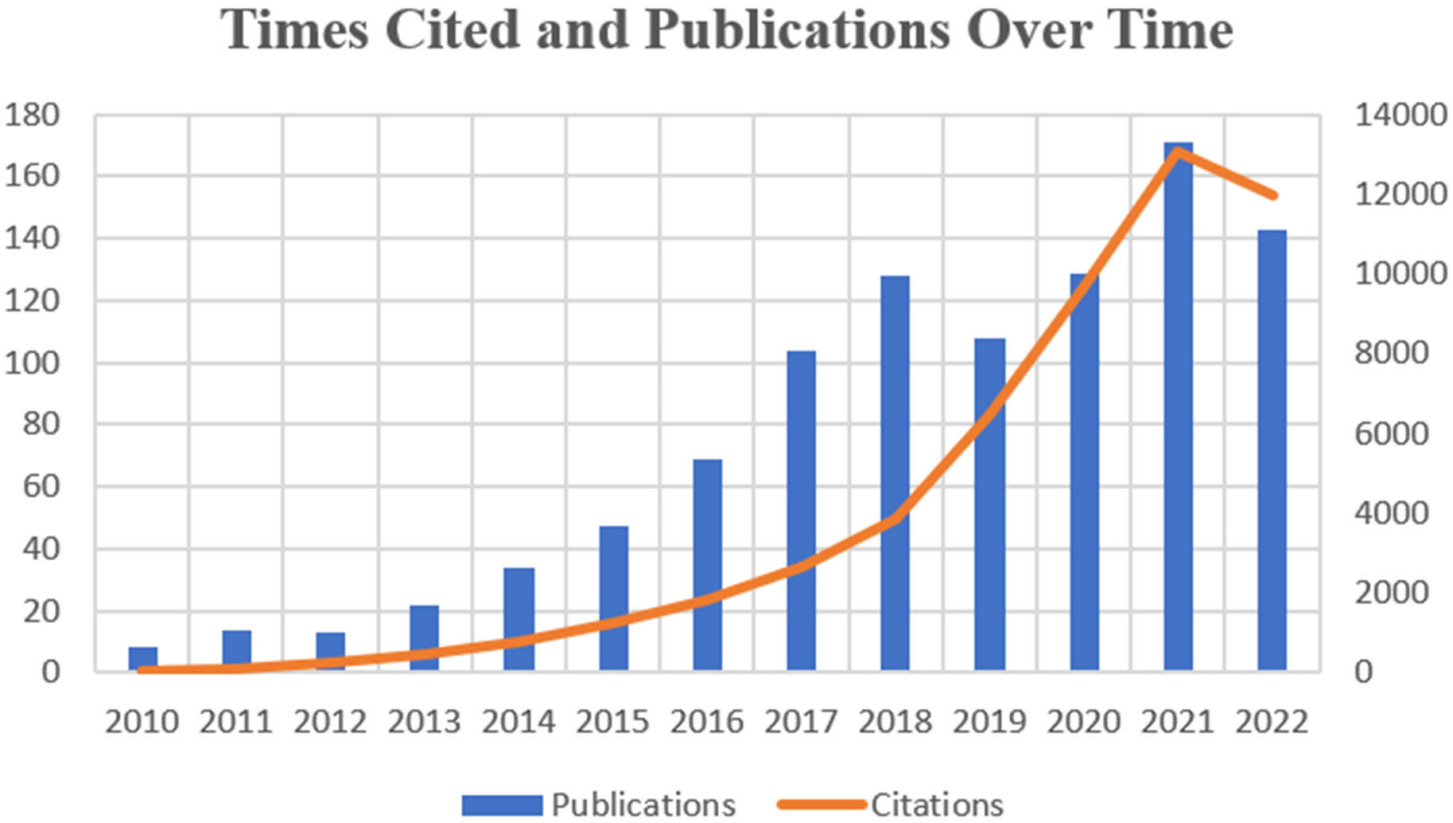
Shows trends in global publication volume and citation counts.

The number and its changes of research paper published in different periods can help reflect the prevalence and developmental trend of research in a specific field. We collected and sorted 990 publications which came out in the last 12 years focusing on exosome in the field of prostate cancer. **Figure 2** shows the variation in global trends of publication outputs and citations. In 2010, there was merely 8 papers published in this field, while in 2017, the publication number firstly surpassed 100. During the last 6 years, the average annual publications remained above 100. And 2021 witnessed both the largest publication number of 172 and the highest citation frequency of 13146. On the whole, the global cited times is growing at a skyscraping rate, exceeding drastically from 31 to 13084 at the peak. Until the search date, the total literatures have attained an overall H-index of 110.

### 3.2 Country/region analysis

**Figure 3:**
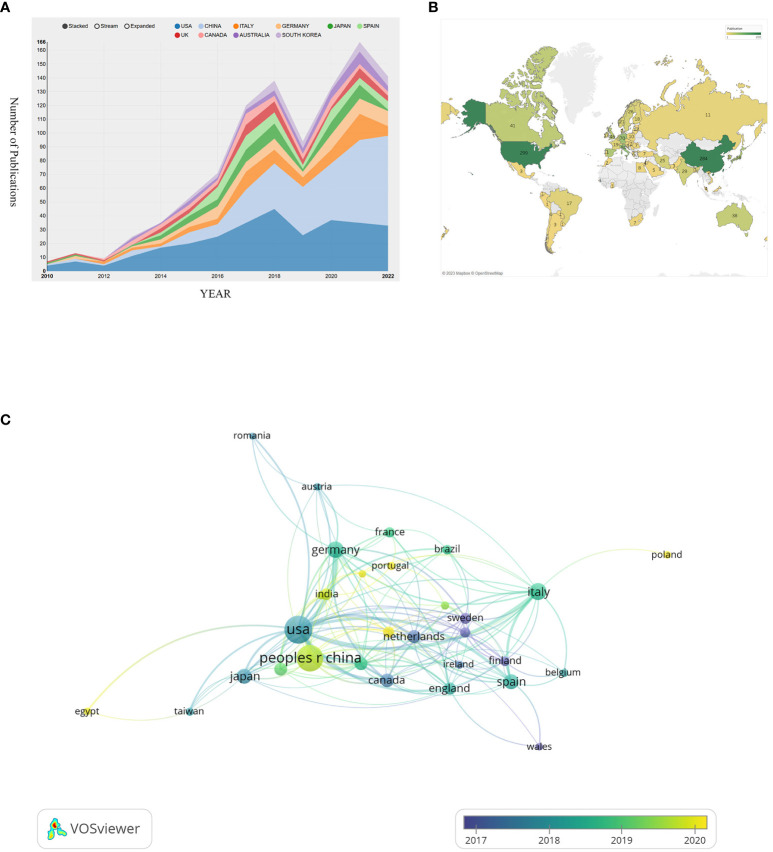
**(A)** Visualizes the annual change in the volume of publications. **(B)** Visualizes a geographical map of contributions of different countries/regions. **(C)** Visualizes the collaborative connection between countries/regions.

To present a more vivid analysis, we have conducted a geographical visualization of publications on prostate cancer-associated exosome. As shown in **Figure 3B**, the research in this area is mainly concentrated in North America, East Asia, West Europe and the Oceania. In **Table 1**, we have concluded the top 10 countries whose publication number rank the highest and attached their relevant information. USA (occupied the first place with total publication of 299), China (284) and Italy (80) have the maximum publications. In terms of Average citations per paper, South Korea, Australia and Canada rank the top three, albeit not so many publications. The H-index and TLS of USA are way ahead, 71 and 215 respectively, suggesting that its cooperation with countries is the broadest and most relevant.

**Table 1:** Summarizes the top 10 countries with the highest volume of publications.

Rank	Country/Region	Publications	Total Citations	Average citation per paper	H-index	TLS
1	USA	299	22137	74.04	71	215
2	CHINA	284	14123	49.73	56	81
3	ITALY	80	6890	86.13	28	78
4	GERMANY	70	7492	107.03	31	80
5	JAPAN	58	7000	120.69	29	31
6	SPAIN	54	6928	128.3	29	51
7	UK	46	6312	137.22	26	52
8	CANADA	41	5742	140.05	26	55
9	AUSTRALIA	38	5649	148.66	20	55
10	SOUTH KOREA	37	5629	152.14	20	28

All the countries/regions in this field fully cooperate with each other and have complex connections. We use VOSviewer to analyze and visualize this connection network. As shown in **Figure 3C**, USA and China occupy the most center location and have the maximum cooperation number of 34 times, with the strongest connection between them. This indicates that they have such sufficient coordination in exploring this field. While the cooperation frequency between USA and German, USA and Italy are 23 and 19 respectively, ranking the following two. The node’s color can represent the time order sequence when they started the research. It’s not hard to find that European countries such as Norway, Finland, Netherlands and Sweden, whose relevant nodes are purple, searched this field at the earliest time. Compared with them, the yellow nodes are represented the latest years. In comparison to USA whose average research publication year is 2017.87, China (2019.6), India (2019.71), and Iran (2020.04) are up-and-comers. Though China started this area late, China’s total publication quantity exceeded USA and became the most published country in this area (**Figure 3A**).

### 3.3 Institutions and funding agencies analysis

**Figure 4:**
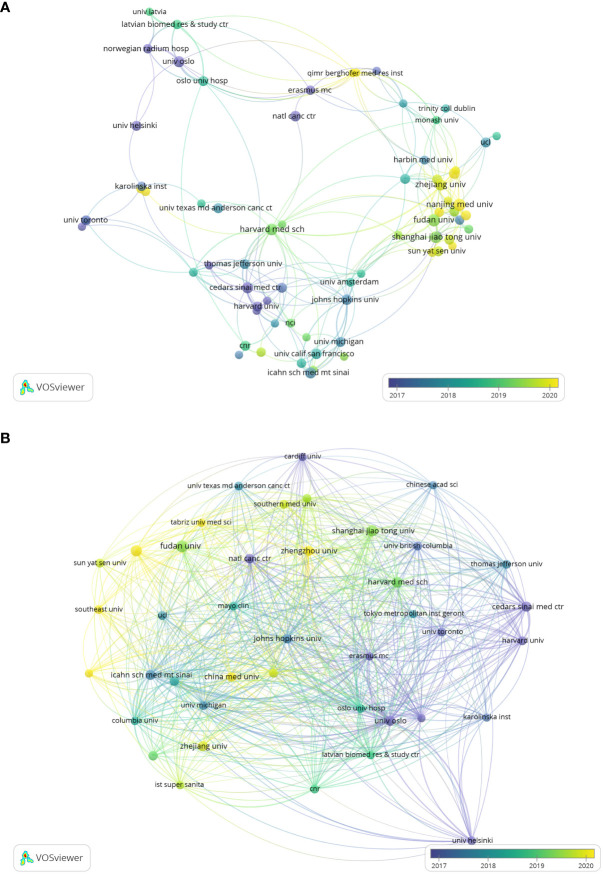
**(A)** Visualizes co-authorship between research institutions. **(B)** Visualizes the citation relationships between research institutions.

As shown in **Figure 4A**, we analyzed the co-authorship of literatures. Three institutions that conducted research in this field earlier are Harvard Medical School, Cedars Sinai Med Ctr, Johns Hopkins University, are located at the core place of the co-authorship analysis. They have the highest TLS, which are 76, 65 and 62 respectively. The purple nodes mean that these institutions started this area research in earlier period, taking Cedars Sinai Med Ctr as an example. Comparing with these European or American institutions whose nodes are mostly purple, Chinese institutions are presented with much green or yellow nodes, which indicate that China’s late start.

**Figure 4B** demonstrates the citation relationships between research institutions, we can find that the most strengthened connection occurred in University Oslo, Oslo University Hospital and Erasmus University Rotterdam, with the highest TLS, in proper order 1718, 1367, 1050.

As shown in **Table 2**, among the top 15 publication research institutions all over the world in this field, USA owns 7 of them, while China has 5. Additionally, Norway, Spain and Italy each have one institution involved. Harvard University, University of California System, Harvard Medical School have the maximum number of publications, 33,32 and 26 respectively. Institution with the highest total citations (6702) and the highest average citations per paper (372.33) is University of Oslo, University of California System and Harvard University just fall behind with total citations of 5988 and 5966. In this field, Harvard University has the earliest average publication year which takes place in 2013.36, while the average publication year of most of Chinese institutions is around 2019, nearly 6 years later.

**Table 2:** Summarizes the top 15 research institutions in terms of publication volume.

Rank	Institution	Country	Documents	Total Citations	Average Citations per paper	Average Publication Year
1	HARVARD UNIVERSITY	USA	33	5966	180.79	2013.36
2	UNIVERSITY OF CALIFORNIA SYSTEM	USA	32	5988	187.13	2018.38
3	HARVARD MEDICAL SCHOOL	USA	26	5056	217.77	2019.2
4	UNIVERSITY OF TEXAS SYSTEM	USA	23	5666	246.35	2017.8
5	SHANGHAI JIAO TONG UNIVERSITY	China	19	575	30.26	2019.31
6	FUDAN UNIVERSITY	China	18	894	49.67	2019.33
7	UNIVERSITY OF OSLO	The Kingdom of Norway	18	6702	372.33	2016.62
8	NANJING MEDICAL UNIVERSITY	China	17	338	19.88	2020.18
9	NATIONAL INSTITUTES OF HEALTH NIH USA	USA	17	4839	284.65	2017.83
10	CIBER CENTRO DE INVESTIGACION BIOMEDICA EN RED	Spain	16	3965	247.81	2017.33
11	CONSIGLIO NAZIONALE DELLE RICERCHE CNR	Italy	16	3781	236.31	2018.82
12	CHINESE ACADEMY OF SCIENCES	China	15	3955	263.67	2019.83
13	ICAHN SCHOOL OF MEDICINE AT MOUNT SINAI	USA	15	4446	296.4	2017.62
14	JOHNS HOPKINS UNIVERSITY	USA	15	4412	294.13	2017.58
15	ZHENGZHOU UNIVERSITY	China	15	579	38.6	2020.29

**Figure 5:**
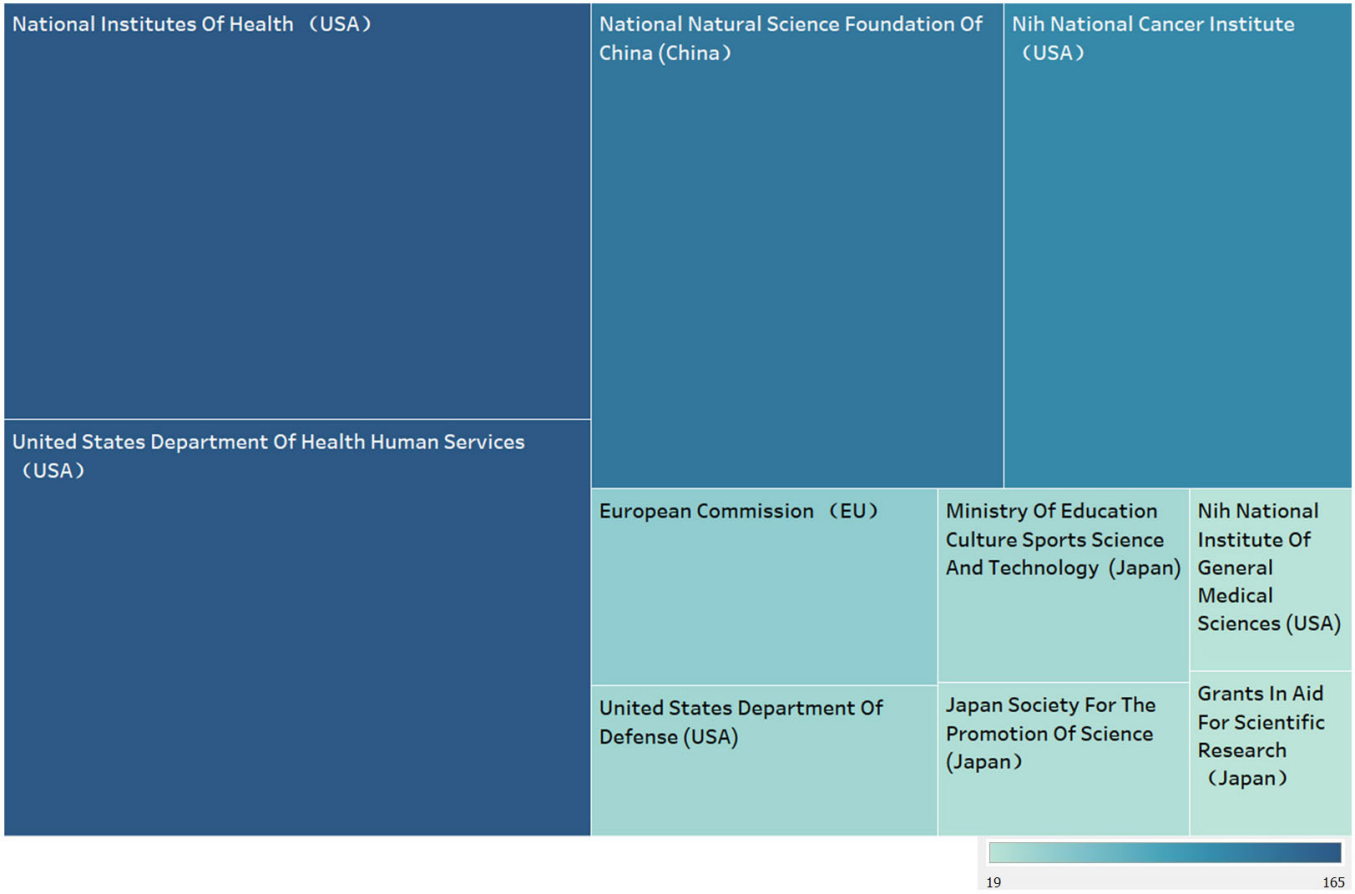
Shows the top 10 funding agencies in terms of publication volume.

**Figure 5** shows the top 10 funding agencies in terms of publication volume, including 7 American agencies and 3 Japanese ones. Amongst all these agencies, National Institutes of Health (USA), United States Department of Health Human Services (USA), National Natural Science Foundation Of China (China) relying on publication of 165, 165, 135 respectively head the table. USA, known as the biggest contributor, tops the chart, while China also takes great percent albeit one institution.

### 3.4 Journal analysis

**Figure 6:**
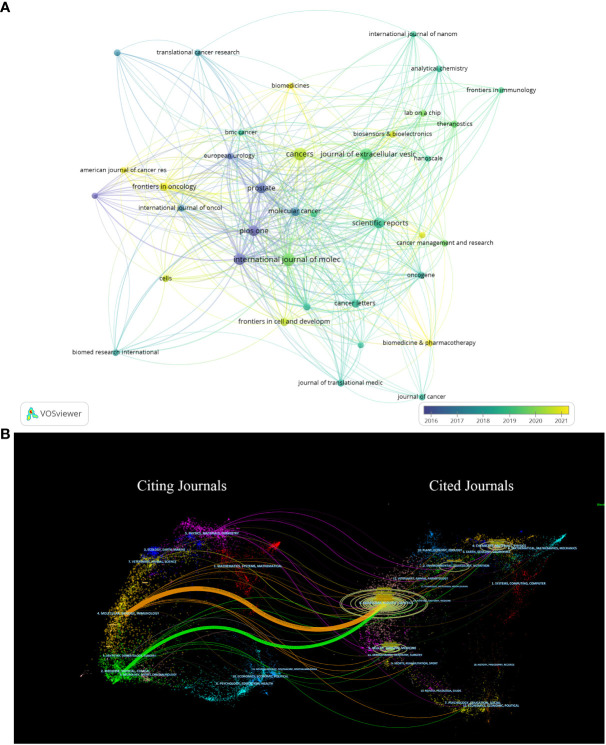
**(A)** Shows the citation relationships between journals. **(B)** Is the journal dual-map produced through CiteSpace.

**Figure 6A** shows the citation relationships, of all the 365 journals which have all published literatures of this field, Prostate, Plos One, Journal of Extracellular Vesicle are at the center. Since the color of the nodes can reveal the time when papers are published, we can calculate and thus find the average publication year of Prostate and Plos One are before 2017, and the yellow nodes which stand for the journals that show solicitude for the research of this field recently, involving Frontiers in Oncology, American Journal of Cancer Research, Frontiers in Cell and Development, Biomedicines, Biosensors and Bioelectronics, Biomedicine and Pharmacotherapy, whose average publication year are behind 2020.

**Figure 6B** vividly displays the citation relationships between journals by using a journal dual-map, with the thickest green and yellow lines representing the strongest citation relationships in this field. These thick lines suggest that literatures published on Molecular/Biology/Immunology and Medicine/Medical/Clinical are usually cited by Molecular/Biology/Genetics or Health/Nursing/Medicine.

**Table 3** summarizes the relevant information of these ten journals, among which USA and Switzerland each have three journals. International Journal of Molecular Sciences (25), Cancers (25), Journal of Extracellular Vesicles (26) have the most publications and Journal of Extracellular Vesicles takes the top spot in term of citations which reaches 4685 times. Furthermore, Molecular cancer, Journal of Extracellular Vesicles, European Urology with much high IF of 41.444, 17.331 and 11.414 carry a lot of clout in this field.

**Table 3:** Summarizes the journals with the highest publication volume.

Journal	Country	Publications	Citations	Total link strength	CR	IF
oncotarget	USA	27	2072	568	NA	NA
international journal of molecular sciences	SWITZERLAND	36	924	418	Q1	6.208
plos one	USA	23	1798	408	Q2	3.752
cancers	SWITZERLAND	36	764	377	Q1	6.575
journal of extracellular vesicles	UK	28	4685	363	Q1	17.331
prostate	USA	23	1158	340	Q2/A3	4.012
european urology	EU	7	1031	310	Q1	24.344
biochimica et biophysica acta-reviews on cancer	NETHERLANDS	9	462	266	Q1	11.414
frontiers in oncology	SWITZERLAND	13	154	264	Q2	5.738
molecular cancer	UK	14	1892	260	Q1	41.444

### 3.5 Author and co-cited author analysis

**Figure 7:**
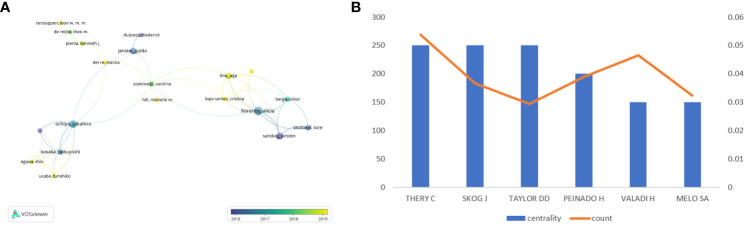
**(A)** Visualizes the collaborations of the leading authors in the field. **(B)** Shows the top 6 authors with the highest centrality in the co-citation analysis.

Different researchers pay close attention to quite different point in this field, as shown in **Figure 7A**, researchers are divided into four groups, separately led by Kosaka Nobuyoshi, Jenster Guido, Line Aija and Llorenta Alicia. It is Ochiya Takahiro and Soekmadji Carolina that make contribution to connect these four groups to a whole.

As shown in **Figure 7B**, we analyzed the information of six authors with the highest centrality. Among them, Thery C, Skog J and Taylor DD have the highest centrality in comparison with others.

**Table 4** summarizes the information of authors whose publications and co-citation rank the top ten. Liorenta A not only has the maximum publications and citations, but also has the highest H-index. Li Y (16) and Ochiya T (14) also have very high publications. Besides, Ochiya T has the citation of 5500 and average citations per paper of 392.86 high; Di Vizio D, who is just a bit behind, has 4429 publications with average citations per paper of 402.64; which indicates their far-reaching implications.

**Table 4:** Summarizes the 10 authors with the highest publication volume.

Rank	Author	Publication	Total citations	Average citations per paper	H-index
1	Llorente A	17	6702	394.24	15
2	Li Y	16	861	53.81	9
3	Ochiya T	14	5500	392.86	13
4	Deep G	13	646	49.69	12
5	Wang L	13	1382	106.31	9
6	Di Vizio D	11	4429	402.64	9
7	Fais S	11	601	54.64	8
8	Kato T	11	338	30.73	8
9	Kim J	11	309	28.09	8
10	Languino LR	11	3852	350.18	9

### 3.6 References and co-cited references analysis

**Figure 8:**
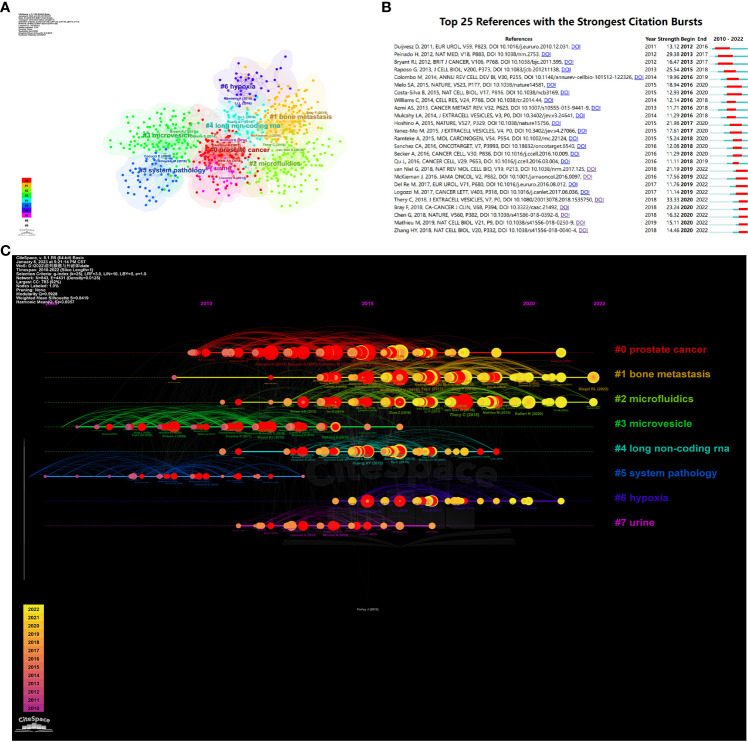
**(A)** Visualizes the cluster analysis for references. **(B)** Shows the top 25 references with the strongest citation bursts. **(C)** Shows the timeline of the references.

We conducted a co-citation analysis on 990 publications included in this study using VosViewer and CiteSpace. As shown in **Figure 8A**, the clustering analysis of reference presents that the top 8 popular research directions combine and connect with each other tightly, with K=9, Modularity Q=0.5928, and Weighted Mean Silhouette S=0.8419, indicating excellent clustering effect and network homogeneity.

Moreover, we visualized the timeline analysis of reference on the basis of the clustering analysis. As shown in **Figure 8B**, widely concerning problems in previous time comprised microvesicle and long non-coding RNA. However, semen, hypoxia, bone metastasis and microfluidics came into the eyesight of researchers currently and became research hotspots of this field.

We sorted out the top 25 references with the strongest citation bursts, as shown in **Figure 8B**. Amongst them, the paper written by Duijvesz D and published in European Urology burst at the earliest time, in 2016. Thery C’s paper named “Minimal information for studies of extracellular vesicles 2018 (MISEV2018): a position statement of the International Society for Extracellular Vesicles and update of the MISEV2014 guidelines” has the maximum burst strength of 33.33 and burst in 2022, obviously revealing the research trend of this area.

### 3.7 Keywords analysis

**Figure 9:**
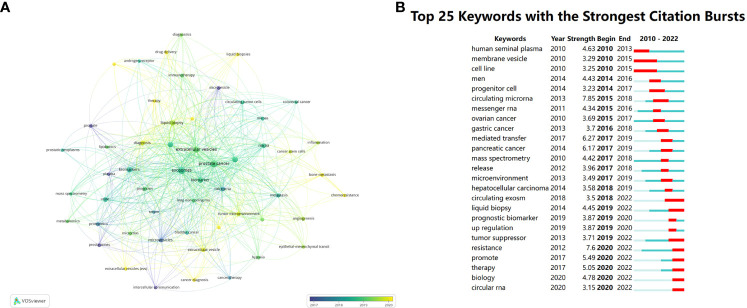
**(A)** Visualizes the co-occurrence analysis of author keywords. **(B)** Summarizes the top 25 keywords with the strongest citation bursts.

We conducted a co-occurrence analysis on author keywords, of the total 1919 keywords, **Figure 9A** displays 54 main keywords. The color of nodes can indicate the order of research time, which clearly reminds us of the tidal current and future orientation of research in this field. Prostate, plasma and prostasomes are the concerns of former research, which can be recognized as purple nodes. Then the study began to focus on biomarkers, exosome and prostate cancer, which are at the core place of the map of keywords. The slight yellow nodes indicate recent hot academic topics, demonstrating that scientific workers turned their eyes to diagnosis, radiotherapy, drug delivery, liquid biopsies, therapy, extracellular vesicles, cancer diagnosis, tumor-derived exosome, tumor environment, bone metastasis, chemoresistance.

Using Burst Detection of CiteSpace, we analyzed 25 keywords with the strongest citation outbreaks. The burst keywords in earlier period consist of human seminal plasma, membrane vesicle and cell line, stronger burst happened to circulating microRNA and mediated transfer with strength of 7.85 and 6.27. However, current burst appears at resistance, promote, therapy and biology. (**Figure 9B**)

## 4 Discussion

In the current rapid development of science and technology, millions of researchers are faced with difficulty in selecting a new project among numerous choices. At the same time, they also created myriad achievements in latest researches, which filled in the gap of a specific subject, not only trying to answer the questions which were not focused before but equally pivotal, but also striving to construct connections between different projects. In face front of such countless literatures and researches, bibliometric analysis, an extensively used method which aims at analyzing total papers in one certain field, offers a very convenient and quick way for researchers to get basic understanding of a new field. It greatly helps researchers analyze the history, previous achievements and the most cutting-edged research interests that have not been concentrated on before. Generally, grasping the current research trend is a necessity for scientific research personnel. Thus, bibliometric analysis, the research we have done, exactly met the need to providing materials to lead the selection of future research and spark inspirations by using data analysis and creating visual images.

As shown in **Figure 2**, there’s an increasing tendency in the publication number and the total citation times in the field of exosome in PCa. Although scarcely and barely less than ten papers published in 2010, the publication number grew steadily from 2013 and achieved the peak in 2021.

This was probably because the Nobel Prize in Physiology or Medicine was awarded to scientists who have made outstanding contributions to the field of intercellular vesicular transport regulation mechanisms in 2013, pushing the research of exosome into heat wave. The decline in 2018 and 2019 was probably owing to the Nobel Prize in Physiology/Medicine in 2018 was awarded to American scientist James P. Allison and Japanese scientist Tasuku Honjo for their discovery and contribution to new methods of cancer treatment. As a result, this partially transferred public attention towards exosome which was believed to be a potential star in future medicine. In addition, the exponential growth rates of global citation also demonstrated the popularity and prevalence of this field in recent years.

As shown in **Figure 3B**, countries involved in this field research are majorly developed countries which are quite prosperous, affluent, and tech savvy, while the involved developing countries are still minority. This can partly convey that the research in this field may consume heavy cost and represent a major expenditure of time and effort. Developing countries may have difficulty in paying the cost of these relevant research proceed from their national actual conditions. Another thing deserved to be mentioned is that developed countries like USA, England, Sweden and Canada also have an earlier start in this field, which prevails their keen insight and sharp perception of the unprecedented scientific interests. Despite that China has a late start in this field (China’s average publication year is 2019.6), almost 2 years after USA whose average publication year is 2017.87, China’s contribution to this field is nonnegligible and remarkable. As shown in **Figure 3A**, China’s total publication quantity exceeded USA and became the most published country in this area. With the second highest total publications and citations shown in **Table 1**, as well as the H-index and TLS at the second place, China has grown up to be a powerful and influential country in this area research, revealing great potential to have long-lasting innovations and achievements in the following years. Other East Asia countries also present passion and strength in researching this area, with high average citation per paper in Japan (120.69) and South Korea (152.14) shown in **Table 1**. There will be a brighter future in seeing more findings coming from East Asia with their intimate cooperation.

We also conducted research focusing on contributions of top institutions and funding agencies. The top three institutions which have the earliest start to carry out researches in this field, Harvard Medical School, Cedars Sinai Medical Center (Cedars Sinai Med Ctr), Johns Hopkins University, are all in the lead in USA academic community. And as shown in **Figure 4B**, Johns Hopkins University took over a central position in the whole field, which mains a perfect choice for researchers to get previous achievements. Besides, USA has the most institutions with the maximum publications at the same time. It’ s quite evident that USA has significant influence and is worthy of scientists’ and institutions’ attention. These institutions should be given priority when considering cooperations. As mentioned above, China has commanded our attention in recent years for its rapid development and great progress. A large amount of Chinese institutions such as Fudan University and Zhejiang University have made cooperations with American top institutions now.

Notably, it’s equally pivotal for researchers to quickly find proper journals which contains cutting-edged publications in a certain field. By means of VOSviewer and CiteSpace, we analyzed the journals and citations in the field of exosome in prostate cancer. Molecular cancer, Journal of extracellular vesicles and European urology stand out accounting for their high publications and citations. These journals perfectly satisfy researchers’ need and sight. Additionally, they can know which journals have tendency to accept papers in this area and probably obtain guidance to publish their documentary with great influence.

Thery C and Skog J have the highest centrality (**Figure 7B**), which is sufficient to illustrate their leadership roles in the field of exosomes and prostate cancer research.

Thery C, as an early pioneer in exosomal research, has led a research team investigating exosomes and tumor growth. She was the first to describe the physical properties of Exosomes, defining them as specific populations of secreted vesicles, and summarized their biological effects, particularly their role in immune system responses (27, 28). Thery discussed the potential of secreted vesicles as intercellular messengers. Moreover, different cells mediate specific secretion of Exosomes and uptake of their cargo, indicating the presence of specificity (26, 29). Additionally, Thery C developed commonly used methods for the isolation and purification of Exosomes from various sources, and discussed approaches to evaluate the purity and homogeneity of purified EV preparations (30, 31). These methods have provided convenience for studying the properties and specific mechanisms of action of exosomes. Furthermore, in recent articles, Thery C explored the role of exosomes in normal physiological and pathological processes, particularly in communication between tumor cells and the establishment of the tumor microenvironment (28, 32).

Skog J is a renowned leader scientist in the field of exosomes and has been at the forefront of groundbreaking discoveries regarding the crucial role played by exosomes and other microvesicles as cellular messengers in disease dissemination. Dr. Skog has demonstrated that tumor-derived exsomes RNA mutations can be detected in serum and other bodily fluids, offering a heterogeneous biomarker for tumor diagnosis (33). His team has also made significant contributions to liquid biopsy techniques for prostate cancer, detecting valuable RNA, DNA, and protein biomarkers in active cell-secreted exosomes, enabling precise diagnostics and aiding clinicians in monitoring cancer progression and determining each patient’s unique genetic composition of the disease. These contribute to our understanding of the leadership roles of Thery C and Skog J in the research on exosomes.

We carried out co-citation analysis (**Table 5**), cluster analysis (**Figure 8A**), timeline analysis (**Figure 8C**), and burst detection on the references (**Figure 8B**). In recent years, the focus can be summarized as the bone metastasis of prostate cancer in relation to exosome, the role of exosome in drug delivery in prostate cancer, the relationship between exosome and tumor suppressors, as well as the association between exosome and drug resistance (**Figures 8**, **9**).

**Table 5:** Summarizes the 10 publications with the highest co-citation counts.

Title	Journals	First author	Year	citations
Minimal information for studies of extracellular vesicles 2018 (MISEV2018)	JOURNAL OF EXTRACELLULAR VESICLES	Thery C	2018	3412
Secretory Mechanisms and Intercellular Transfer of MicroRNAs in Living Cells	JOURNAL OF BIOLOGICAL CHEMISTRY	Kosaka N	2010	1407
Current knowledge on exosome biogenesis and release	CELLULAR AND MOLECULAR LIFE SCIENCES	Hessvik NP	2018	1159
Extracellular Vesicles in Cancer: Cell-to-Cell Mediators of Metastasis	CANCER CELL	Becker A	2016	990
Extracellular vesicles in cancer - implications for future improvements in cancer care	NATURE REVIEWS CLINICAL ONCOLOGY	Xu R	2018	696
Characterization of human plasma-derived exosomal RNAs by deep sequencing	BMG GENOMICS	Huang XY	2013	679
Suppression of Exosomal PD-L1 Induces Systemic Anti-tumor Immunity and Memory	CELL	Poggio M	2019	552
Lipids in exosomes: Current knowledge and the way forward	PROGRESS IN LIPID RESEARCH	Skotland T	2017	532
Changes in circulating microRNA levels associated with prostate cancer	BRITISH JOURNAL OF CANCER	Bryant RJ	2012	522
Exosomes from human saliva as a source of microRNA biomarkers	ORAL DISEASES	Michael A	2010	488

Although prostate cancer is the second most common diagnosed cancer in males, localized or regional lesions do not directly lead to death. However, the prognosis for metastatic disease is usually not optimistic, which may be the main reason why prostate cancer ranks fifth in global cancer-related mortality. It can be inferred that the ability of prostate cancer to acquire metastasis is the most lethal part of its disease progression. The bone is a relatively common site of metastasis, with 70% of advanced prostate cancer patients found to have bone metastasis (34).

Normal prostate epithelial cells are tightly adhered to neighboring cells and the surrounding extracellular matrix (ECM). However, malignant prostate cells have reduced adhesion and detach from adjacent cells and ECM, facilitating migration and invasion. Therefore, tumor cells in advanced prostate cancer can detach, migrate into the bloodstream, and enter the bone marrow through the lymphatic or hematogenous system, leading to additional bone metastasis. The growth of prostate cancer after metastasis initially relies on the establishment of the premetastatic niche, which requires factors that can be produced endogenously by osteoclasts or generated, transmitted, and secreted by cancer cell-derived exosomes (35, 36). Factors such as interleukins, bone morphogenetic proteins, VEGF, and chemokines secreted by primary PCa cells have been shown to play a role in promoting tumor metastasis and progression (25, 37–40). Exosomes have been shown to carry tumor-related molecules that play a role in cancer and premetastatic niche (41, 42). Additionally, research has indicated the importance of the bone microenvironment in promoting tumor colonization and proliferation, similar to the tumor cells themselves (43).

It is noteworthy that, in line with the seed and soil hypothesis of tumor development, the tumor microenvironment constructed as a result complements and influences the occurrence and progression of prostate cancer. The primary PCa needs to undergo a lengthy process of bone remodeling to develop into bone metastasis, which involves metastatic cancer cells, as well as intrinsic bone cells such as osteoblasts, osteoclasts, and stromal cells (44). The metastatic cancer cells are partially in a dormant state, awaiting suitable conditions for activation, and their role is to promote new bone formation. Therefore, the activation of osteoclasts and osteoblasts during bone remodeling is key to promoting tumor growth. Research has shown that cancer cells secrete more than 10 times the amount of exosomes compared to normal cells. Moreover, exosomes derived from tumors promote intercellular communication through growth factors, chemokines, miRNA, and other small molecules (45, 46). Tumor-derived exosomes play an important role in mediating intercellular communication during the bone metastasis process of osteoblastic cells. Osteoblasts specifically take up the secreted exosomes from PCa cells, undergo reprogramming, and subsequently promote PCa cell homing and local proliferation (47). Furthermore, human bone marrow stromal cells can also take up exosomes derived from PCa cells, leading to changes in transcriptional level and signal transduction, promoting cancer cell metastasis (48). This highlights the importance of establishing a signaling network in the tumor microenvironment.

In bone metastasis, tumor-derived exosomes also have an osteogenic differentiation-inducing effect on the local mesenchymal cell pool. In addition to tumor cell-derived exosomes, exosomes derived from other cells also participate in this process. For example, osteoblast-derived exosomes can regulate the proliferation of cancer cells in the metastatic microenvironment (49, 50). However, the molecular mechanisms underlying PCa bone metastasis formation are not fully understood. Specifically, it is unclear how certain substances present in the microenvironment, such as cellular factors and immune molecules, are secreted into the ECM from tumor cells, osteoclasts, fibroblasts, etc., whether through exocytosis, exosomes, or other forms. Exosomes are typically regarded as carriers of proteins, lipids, and RNA, capable of targeted delivery to specific cells and acting on membrane receptors to initiate signal transduction.

However, the role of exosomes in this pathological process is not well elucidated. Therefore, we believe that exosomes may play a role in prostate cancer bone metastasis. Research on the specific contributions of exosomes from various sources to the tumor ECM could potentially improve the accuracy of metastatic prostate cancer diagnosis and address the current issue of overdiagnosis. The composition and characteristics of exosomes derived from different cell sources exhibit heterogeneity. Revealing the substances secreted by tumor cell-derived exosomes, compared to those released by bone-related cells and fibroblasts into the ECM, may contribute to targeted therapy for PCa.

Exosomes are small particles which can be seen in and secreted by all cells in the organism, thus are a heterogeneous population of lipid bilayer membrane-enclosed vesicles. The promising prospects of exosomes as drug delivery vehicles are attracting the attention of more researchers. The characteristics of exosomes themselves align with the requirements of targeted drugs for cancer treatment: high targeting specificity, ability to traverse cell membranes, immune tolerance, multifunctionality, and feasibility for large-scale production. Exosomes exhibit high targeting specificity, as they can achieve precise targeted delivery through interactions between the heterogeneous surface proteins of exosomes and the matching proteins on target cells (26). The molecular regulation of fusion between different cell-derived exosomes and target cell plasma membranes varies and is likely dependent on the organism, cell type, and exosome subtype. Currently, there are no specific targeted drugs that have been proven effective for the treatment of prostate cancer, which exhibits high heterogeneity. There may be differences in tumor characteristics and gene expression between different patients or within different lesions of the same patient. This can result in a lack of available options for some patients with advanced prostate cancer who may be resistant to mainstream treatment drugs or methods. Therefore, under the premise of understanding the mechanism of exosome action, it is possible to artificially design vesicles that assemble RNA with therapeutic effects, which can improve the limitations of existing personalized medicine. Additionally, the ability of exosomes to traverse biological membranes, including cell membranes and the blood-brain barrier (26), demonstrates their feasibility and effectiveness in delivering drugs to target cells or tissues. Exosomes are considered a natural drug delivery system with low immunogenicity and toxicity, and they may also participate in immune responses. Compared to other nanocarriers such as nanoparticles or liposomes, exosomes are more readily accepted by the body, reducing immune reactions and improving biocompatibility. In addition to serving as drug carriers, exosomes can also exert other functions during drug delivery. For example, they may influence cell signaling and gene expression. This multifunctionality enables exosomes not only to transport drugs but also to modulate disease environments. In recent years, with the advancement of technology, exosomes can be derived from various cell sources including cell lines cultured *in vitro* and endogenous sources *in vivo*. This provides diverse options for exosome production and holds the potential for achieving large-scale production to meet the demands of drug delivery. Despite the existing limitations (51), an increasing number of new technologies such as “exosomes for protein loading via optically reversible protein–protein interactions” (EXPLORs) (52) and cellular nanoporation (53) should be implemented to facilitate the loading of exogenous cargoes into exosomes. In conclusion, due to their low cytotoxicity, ability to maximize the bioavailability of drugs, and precise targeted homing specificity (26, 54), there has been an increasing number of clinical trials confirming the therapeutic potential of exosomes. They have been successfully applied in the treatment of chronic kidney disease (55), non-small cell lung cancer (56), colon cancer (57), breast cancer (58), and more. We believe that this will also provide help in the treatment prospects of prostate cancer.

Exosomes have a unique potential role in anti-tumor effects. Factors mediated by exosomes are involved in cell communication within the tumor microenvironment (TME), thus promoting tumor initiation and metastasis (59–61). Multiple studies have revealed that the TME affects tumor behavior by altering the malignant behavior of tumor cells (62). Exosomes, located within the TME, influence tumor progression through various tumor-promoting pathways such as angiogenesis, metastasis, and hypoxia induced by epithelial-mesenchymal transition (EMT) (62). As depicted in **Figure 9**, these have been research hotspots. Apart from the well-known role of gene mutations reaching a certain threshold in the initiation of tumors, the functional changes of exosomes in the TME also contribute to tumor initiation (62–64). In the reshaping of the TME, the promotion of cancer cell invasion may be attributed to the activation of exosomes leading to the release of tumor progression factors, including growth factors (65), RNA, and proteins (66). It may also be due to EMT, in which scientists have observed an increase in the release of mesenchymal markers associated with exosomes (67). In the field of prostate cancer, exosomes-mediated cooperation of miR-21-5p, miR-100-5p, and miR-139-5p has been shown to promote the progression and metastasis of PCa (68). Previous studies have indicated that the removal of exosomes from circulation can inhibit tumor progression (69). Tumor-derived exosomes can enhance cancer cell migration and invasion by downregulating tumor suppressor factors, as observed in breast cancer cells (70). Moreover, dendritic cells (DC cells), as the most effective antigen-presenting cells in the body, play a central role in initiating and regulating innate and adaptive immunity in the tumor microenvironment. They have the ability to present tumor-associated antigens on MHC molecules, provide co-stimulatory molecules or soluble factors to induce anti-tumor cellular responses (71). Immunogenic cell death can enhance tumor antigen exposure and promote tumor killing (72–74). Therefore, exosomes derived from dendritic cells (DCs) are potential candidates for specific cancer therapy, as they have been proven to induce immune system activation (75). Tumor-derived exosomes (TDEs) can theoretically also serve as therapeutic carriers for *in situ* DC activation through tumor microenvironment infiltration. Anti-tumor drugs based on this principle have been developed (76). This provides a new approach to inhibit the progression of prostate cancer. In addition, drug-loaded exosomes can improve the anti-tumor outcomes of chemotherapy drugs, such as paclitaxel-loaded exosomes for the treatment of various cancers including prostate cancer and lung cancer (77, 78).

Exosomes have been found to be closely related to tumor therapy resistance (59–61, 79). Exosomes may be hijacked in cancer chemotherapy. Its mechanism is related to the process of epithelial-mesenchymal transition (EMT) (80–82). Exosomes play a central role in multiple steps of EMT, ranging from the generation of invasive phenotypes to distant metastasis (83, 84). Resistant cancer cells can package chemotherapy drugs into exosomes and shuttle anticancer drugs out of tumor cells (85). Exosomes can carry and exchange genetic material with target cells, and resistant cancer cells utilize this to confer resistance on sensitive cells (86, 87). Additionally, TDE can induce resistance by promoting pathways of apoptosis inhibition (88–91). Cancer stem cells (CSC), which are also critical members in tumor development, maintain self-renewal and certain characteristics, as well as participate in cell migration, differentiation, and proliferation, through the transfer of their derived exosomes carrying cargo. Therefore, they enhance resistance to cancer treatment (92). With the advancement of cancer therapy, the emergence of resistance poses new challenges for researchers and medical teams. While exploring novel therapies for prostate cancer using exosomes, it is crucial for researchers to also be aware of the potential issue of resistance that may be encountered.

## 5 Conclusion

Through a comprehensive analysis of entire aspects of the PCa associated exosome field, we conducted a bibliometric and scientometric analysis of the research trends and hotspots in the field of exosome related to prostate cancer. USA and China have been in the center of this area with the high citations and publications, which makes us believe their potential in exploring the pathogenesis of exosomes in PCa. Over the past 12 years, the research focus has shifted from the generation and function of exosome to their role in tumor progression and drug therapy. Based on our findings, we predict that the research focus in this field will revolve around the role of exosomes in bone metastasis of prostate cancer, tumor treatment resistance, and the potential for future drug development. Researchers have started to pay attention to the unique properties of exosome in order to explore their potential effects in drug delivery. Accordingly we have sufficient reasons to believe that this field is about to witness more research interests and development prospects.

## 6 Limitations

This bibliometric analysis may have a few limitations. Firstly, we have relied solely on the WOSCC database for literature extraction. While WOSCC is widely regarded as a comprehensive database, it is still possible that some relevant publications may have been overlooked. Furthermore, our analysis may have excluded certain important publications due to restrictions on language and types of publications. Additionally, for the purpose of enhancing the clarity of our visual representations, we have selectively screened and included significant literature in the field for our analysis and mapping.

## Explanation of nouns

1. Cluster Analysis is a data mining technique that uses statistical analysis to divide a data set into categories based on some similarity measure. The purpose of cluster analysis is to allocate data items with similar features to the same group, and data items with different features to different groups. The goal is to organize large amounts of data into meaningful clusters for better understanding and interpretation of the data.
2. Co-citation analysis refers to when two papers appear in the reference directory of a third paper, forming a co-citation relationship. The co-citation analysis method evaluates the similarity of papers by examining the number of times they are cited after publication. If two authors’ papers are cited by the same third author’s paper, then these two authors have a co-citation relationship, and the higher the frequency of their co-citation, the closer their academic relationship. The clustering function of CiteSpace was used for cluster analysis of literature co-citation, and the common themes of similar papers can be mined. The core papers of the co-citation cluster analysis may become research trends and hotspots.
3. Co-occurrence Analysis refers to the analysis of the frequency of occurrence of different entities within the same time or space, in order to evaluate their degree of association. Co-occurrence analysis is a quantitative study of co-occurrence phenomena, to reveal the content associations and knowledge implied by the features. This type of analysis is commonly used in fields such as data mining and social network analysis to discover relationships or patterns and to serve as support for decision-making.
4. Centrality is a metric that measures the proximity of a node in a network to the center of the entire network. By evaluating a node’s centrality, the importance of the node in the network can be determined. In graph theory and network analysis, centrality metrics can identify the most critical node in a graph.
5. Total Link Strength (TLS) refers to the sum of the strength of a node’s relationships with other nodes in a network. This strength can be represented by weights and can be evaluated based on the characteristics of the relationship between two nodes. TLS can be used to assess a node’s influence and centrality in a network.
6. A timeline is a graphical representation of a timeline that displays the sequence of events or historical events. It can help people clearly understand the order of events, the relationship between events and the relative length of each event. Its purpose is to visualize content and present it in a graphic and textual form to assist the reader in understanding.
7. Burst detection is a technique in data mining and information processing that detects sudden growth or events in data. It is commonly applied to time-series data to identify instances where the volume of data significantly increases in a short period and determine the cause of this growth.

## Data availability statement

The original contributions presented in the study are included in the article/**Supplementary Material.** Further inquiries can be directed to the corresponding author.

## Ethics statement

Ethical approval was not required for the study involving humans in accordance with the local legislation and institutional requirements. Written informed consent to participate in this study was not required from the participants or the participants’ legal guardians/next of kin in accordance with the national legislation and the institutional requirements.

## Author contributions

ZZ: Data curation, Formal Analysis, Methodology, Visualization, Writing – original draft. YZ: Conceptualization, Methodology, Software, Visualization, Writing – original draft, Writing – review & editing. HL: Resources, Writing – original draft. WX: Resources, Writing – original draft. TW: Data curation, Writing – original draft. JL: Resources, Writing – original draft. HJ: Conceptualization, Project administration, Writing – review & editing.

## Supplementary material

Supplementary Material 1 Shows the ten journals with the highest TLS in citation analysis.
