# Supplementary figures and images for: Research trends and hotspots in prostate cancer associated exosome: a bibliometric analysis
Source: Front Oncol. 2023 Nov 21;13:1270104. doi: 10.3389/fonc.2023.1270104 (PMC10712200; doi:10.3389/fonc.2023.1270104)

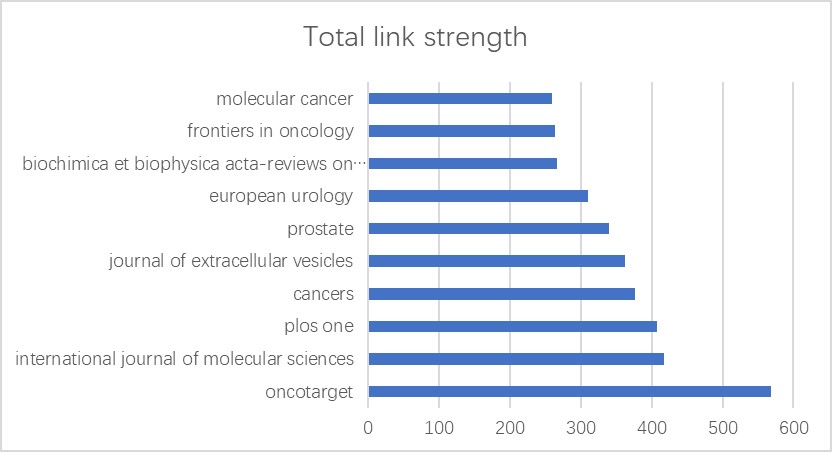

Supplement: Supplementary Material 1 — Shows the ten journals with the highest TLS in citation analysis. [file Image_1.jpg]
